# An In Vitro Assessment of Gutta-Percha Coating of New Carrier-Based Root Canal Fillings

**DOI:** 10.1155/2014/239754

**Published:** 2014-03-06

**Authors:** Raghad Abdulrazzaq Alhashimi, Richard Foxton, Shihab Romeed, Sanjukta Deb

**Affiliations:** ^1^Department of Conservative Dentistry, King's College London Dental Institute, Floor 25, Tower Wing, Guy's Hospital, London Bridge, London SE1 9RT, UK; ^2^Biomaterials, Biomimetics, Biophotonics Research Division, King's College London Dental Institute, Floor 17, Tower Wing, Guy's Hospital, London Bridge, London SE1 9RT, UK; ^3^Department of Restorative Dentistry, King's College London Dental Institute, Floor 25, Tower Wing, Guy's Hospital, London Bridge, London SE1 9RT, UK

## Abstract

The first aim of this paper was to evaluate the push-out bond strength of the gutta-percha coating of Thermafil and GuttaCore and compare it with that of gutta-percha used to coat an experimental hydroxyapatite/polyethylene (HA/PE) obturator. The second aim was to assess the thickness of gutta-percha around the carriers of GuttaCore and HA/PE obturators using microcomputed tomography (**μ**CT). Ten (size 30) 1 mm thick samples of each group (Thermafil, GuttaCore, and HA/PE) were prepared. An orthodontic wire with a diameter of 0.5 mm was attached to the plunger of an Instron machine in order to allow the push-out testing of the gutta-percha coating. Five samples of (GuttaCore and HA/PE) were scanned using **μ**CT. The data obtained were analysed with one-way analysis of variance and Tukey post hoc test. HA/PE obturators exhibited significantly higher push-out bond strength (*P* < 0.001) determined at 6.84 ± 0.96 than those of Guttacore around 3.75 ± 0.75 and Thermafil at 1.5 ± 0.63. GuttaCore demonstrated significantly higher bond strength than Thermafil (*P* < 0.001). **μ**CT imaging revealed that the thickness of gutta-percha around the experimental HA/PE carrier was homogeneously distributed. The bondability and thickness of gutta-percha coating around HA/PE carriers were superior to those of GuttaCore and Thermafil obturators.

## 1. Introduction

It is generally accepted that the outcome of the root canal treatment is positively correlated with the technical quality of the root canal obturation [1]. Previous studies have shown the effectiveness of thermoplasticized core carrier techniques in obtaining three-dimensional root canal fillings in a reduced amount of time compared to the lateral condensation technique [2–4]. Carriers for core-based techniques can be fabricated using different materials; Thermafil small size obturators (up to size 40) (Tulsa Dental Dentsply, Tulsa, OK, USA) are made of Vectra, which is a liquid crystal polymer and larger sizes are made of polysulfone, whereas GuttaCore carriers (Tulsa Dental Dentsply) are made of cross-linked gutta-percha. These materials are coated with alpha-phase gutta-percha.

The experimental carrier made of hydroxyapatite-polyethylene-strontium oxide was recently developed [5]. Most root canal filling materials do not thoroughly obturate the root canal system, leaving some voids either within the root filling material or at their interface with dentine [6]. These voids might harbour bacteria that can multiply when in contact with nutrients via the periapical region or lateral canals [7].

One potential disadvantage of a carrier-based root filling system is denudation of the core with stripping of the gutta-percha coating [8]. Stripping of gutta-percha from the carrier might happen during the insertion of the carriers into the root canal space, particularly in narrow or severely curved canals. This would result in voids and inadequate filling of the root canal space [9]. Previous studies have shown that the most common causes of stripping of the gutta-percha coating are twisting the carrier during insertion into the root canal space and inadequate amounts of sealer placed prior to insertion of the obturators in the root canal [10, 11]. Adhesion between the carrier and gutta-percha coating is therefore an important aspect in the choice of a core-based obturation system and would help avoid stripping of the gutta-percha coating, creating a root canal filling with fewer voids.

Another potential disadvantage of currently available carrier-based obturation systems is that the volume of gutta-percha is not uniformly distributed around the carrier. This might cause stripping of the gutta-percha from the carrier material when the obturator is inserted into the root canal space leading to possible voids [8]. The frictional forces present between the gutta-percha and the root canal walls may create an extrusion effect, whereby the filling material is retained at the orifice of the canal [12]. High-resolution micro-CT is an emerging technology with several promising applications in many different fields of dentistry [13] including endodontics [14, 15]. Previous studies using the *μ*CT have shown the possibility of conducting both volumetric measurements [15] and 3D reconstruction of obturated root canals and their constituents [13].

To date, there have been no studies which have evaluated the strength of the bond between the carrier material and gutta-percha and the volume of gutta-percha around the carrier using *μ*CT. The purposes of the present study were therefore to compare the push-out bond strength between gutta-percha coatings and three types of carrier materials, in particular, Thermafil (Dentsply Tulsa Dental, Tulsa, OK), GuttaCore (Dentsply Tulsa Dental), and an experimental carrier made of hydroxyapatite/polyethylene (HA/PE) and strontium oxide, and to compare the thickness of gutta-percha between GuttaCore and HA/PE systems. The following hypotheses were tested: (i) the adhesion (push-out bond strength) between gutta-percha coating and the test carrier materials is the same and (ii) the thickness of gutta-percha coating around the experimental HA/PE carrier is not different from that of GC carrier.

## 2. Materials and Methods

### 2.1. Push-Out Test Specimen Preparation

The procedure used to fabricate the newly designed carrier has been previously described [5]. In brief, the hydroxyapatite (HA) powder with a mean particle size of 3–5 *μ*m (Plasma Biotal, Derbyshire, UK) was treated with a silane coupling agent solution (A174, Merck KGaA, Frankfurt, Germany) in order to improve the bond strength between the HA particles and the matrix, creating a more stable and durable composite material. Then the silanated HA (20 wt%) was mixed with (70 wt%) low-density polyethylene (Good Fellow Chemical Products, Huntingdon, England) and SrO (10 wt%) as a radiopacifier (Alfa Aesar, Ward Hill, UK) thoroughly and placed in a hopper of a 12 mm single screw extruder. The processing temperature was maintained at 160°C and the speed of the rotary screw was optimized at around 25 rpm. The material was extruded through a die drawn down to different diameters and tapers. Subsequently, the composite carriers were successfully coated with gutta-percha (GP) to form the experimental carrier-based root canal obturator (HA/PE). This process was performed by dissolving the alpha phase of GP in chloroform until a viscous liquid was obtained. A cylindrical rubber tube with a stopper was prepared to receive the composite carrier material. GP was injected using a very fine needle into the tube, creating an evenly distributed layer of GP around the carrier [5].

Three types of endodontic obturators were tested: Thermafil (Dentsply Tulsa, OK), GuttaCore (Dentsply Tulsa), and the experimental hydroxyapatite/polyethylene (HA/PE) obturator as shown in Table 1.

**Figure 1:**
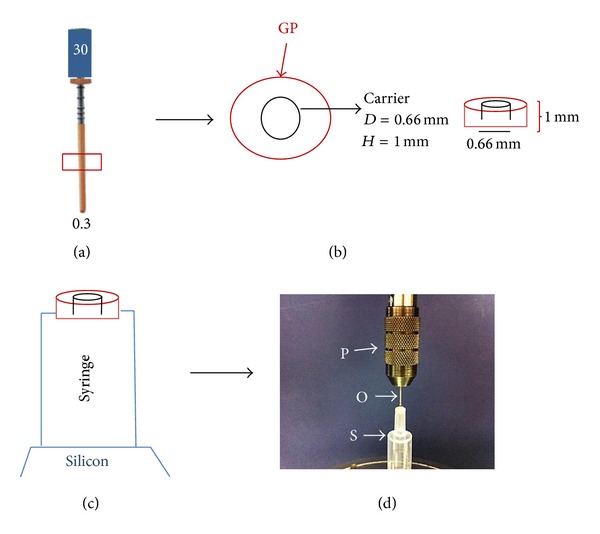
Schematic representation of the push-out test procedure. (a) The middle part of the endodontic obturator is chosen for the test. (b) Slices (height 1 mm and diameter 0.66 mm) are obtained. (c) The slice is fitted into the aperture of a customized syringe. (d) The plunger of the Instron machine is equipped with an orthodontic wire (0.5 mm in diameter) and aligned with the slice to be tested (P = Instron plunger, O = orthodontic wire, and S = syringe holding the slice).

Ten sized 30/.04 with 25 mm carriers from each carrier type were selected. The portion of each carrier coated with gutta-percha was divided into three parts, 5 mm long (coronal, middle, and apical). One slice (1 mm thick) was obtained from the junction point of the coronal portion and middle portion of each carrier using a low speed diamond wafering blade (Isomet; Buehler, Lake Bluff, IL) as shown in Figure 1(a). The diameters of the carriers in the portion used to obtain the slices were measured using a digital calliper (Maplin Electronics, Rotherham, UK) and were found to be 0.66 mm ± 0.04 mm in case of GuttaCore and Thermafil obturators and 0.60 ± 0.02 mm for the experimental HA/PE carriers. Each 1 mm thick slice was fixed to the aperture of a plastic syringe (Terumo, Leuven, Belgium) whose diameter was 1.5 mm in a vertical position using a cyanoacrylate adhesive (Zapit, Dental Ventures of America, Corona, CA, USA) as shown in Figures 1(b) and 1(c). A 0.5 mm round orthodontic wire was attached to the plunger of a universal testing machine (Instron model 5569 A, High Wycombe, UK) in order to load the carrier at a constant crosshead speed of 0.5 mm/min (Figure 1(d)). A silicon-based material (Metrodent, Huddersfield, UK) was used to fix the syringe with its long axis parallel to the long axis of the plunger. On the loading machine, each slice was positioned with the larger side of the carrier segment placed facing the punch tip. Bond failure was considered to be the displacement of the carrier segment from the gutta-percha. Push-out strength data were determined in MPa by dividing the load in Newton by the bonded surface area (SL) in mm^2^. SL was calculated using formula [16]: SL = (*R*_1_ + *R*_2_)*√*(*R*_1_+*R*_2_)^2^ + *h* ^2^, where *π* = 3.14, (*R*_1_) is the apical carrier radius (base), (*R*_2_) is the coronal carrier radius (top), and *h* is the height of the slice.

### 2.2. Microcomputed Tomography (*μ*CT) Specimen Preparation

**Figure 2:**
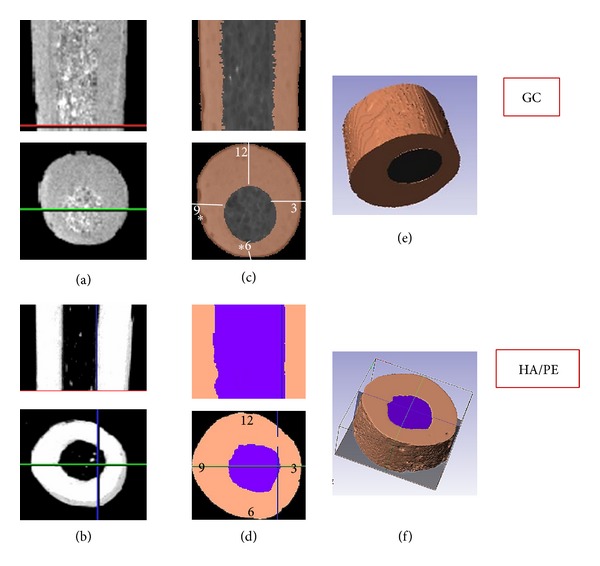
Microcomputed tomography images prepared from the middle third of the GC and HA/PE obturators. (a, b) *μ*CT scans of the GC and HA/PE systems, respectively, showing slice selection from the middle third. (c, d) *μ*CT slices of GC and HA/PE, respectively, segmented by Scan IP using different masks. (e, f) 3D reconstruction of GC and HAPE systems using Scan IP based on voxel density.

Five further samples of GuttaCore (Dentsply Tulsa) and HA/PE (experimental) obturators were prepared from the middle third of the obturators (Figures 2(a) and 2(b)). These obturators were scanned using a GE Locus SP *μ*CT scanner (General Electric, London, ON, Canada) with an X-ray tube voltage of 80 kV and a current of 80 *μ*A. A 0.1 mm aluminium filter was used to attenuate the X-ray source. The specimens were immobilised using cotton gauze and scanned to produce 6.5 × 6.5 × 6.5 *μ*m voxel size volumes. The samples were characterised further by making three-dimensional reconstructions of all the obturators. All scans were imported by Scan IP (Simpleware, Exeter, UK) as a stack of images where a segmentation had been carried out on each individual slice according to the pixel density. Two masks were produced to represent the gutta-percha coating and the carriers. The thickness of GP around its carrier was calculated along nine slices of each sample. Four identical fixed points on each slice were identified for thickness measured in both samples in order to make a comparison between GP-coated GC and HAPE cores as shown in Figures 2(c), 2(d), 2(e), and 2(f). Statistical analysis was performed using GraphPad Prism software version 5 (La Jolla, CA USA). One-way analysis of variance and Tukey post hoc test were used to determine any differences between the groups. The level of significance was set at *P* = 0.05.

## 3. Results

The findings of the push-out strength (MPa) are summarized in Table 2. The mean push-out bond strength of the experimental obturators, GuttaCore and Thermafil obturators, was 6.84 ± 0.96, 3.75 ± 0.75, and 1.5 ± 0.63 MPa, respectively. The push-out bond strength of the experimental carriers was found to be significantly higher (*P* < 0.001) than those of GuttaCore and Thermafil. GuttaCore push-out bond strength was significantly higher than Thermafil (*P* < 0.001).

The *μ*CT scans demonstrated that the volume of gutta-percha in the HA/PE system was more symmetrical and homogenous than that of GuttaCore as shown in Figure 2. The thickness of gutta-percha in the GuttaCore system from four points “3, 6, 9, 12” was 0.376 ± 0.027, 0.164 ± 0.023, 0.357 ± 0.033, and 0.591 ± 0.034 mm, respectively. On the other hand, the HA/PE showed a thickness of gutta-percha coating at “3, 6, 9, 12” o'clock positions to be 0.419 ± 0.03, 0.607 ± 0.042, 0.584 ± 0.039, and 0.535 ± 0.04 mm, respectively. The difference in volume of gutta-percha between GuttaCore system and HA/PE was significant (*P* < 0.05) particularly at the “6” and “9” o'clock positions.

## 4. Discussion

Carrier-based techniques are prone to exposure to the carriers during their insertion into the root canal, which would cause the formation of voids between the root filling material and the root canal walls. Adhesion between the gutta-percha and carrier material is therefore an important requirement to effectively obturate the root canal system. Stripping of gutta-percha from the carrier has been observed by previous researchers in the apical third and in the middle and apical thirds [17, 18]. We developed carriers of different diameters and tapers made of hydroxyapatite-polyethylene (HA/PE) fibres, which could be easily removed should retreatment be necessary and with micromechanical adhesion between the gutta-percha and carrier. This newly designed carrier has been described in a previous paper [5].

To the authors' knowledge this is the first paper in which a push-out test has been used to test the bond strength between the GP coating and the carrier of core-based root canal filling systems. Although the expected push-out values that would prevent gutta-percha stripping off in a carrier-based obturator during the obturation procedure are still unknown, the satisfactory adhesion of gutta-percha to the carrier material may be effective. It is worthwhile clarifying two points regarding the geometry of the carrier and their possible effects on the push-out results. The first issue is related to the presence of a groove in Thermafil and GuttaCore carriers. This groove has been devised to facilitate the retrieval of the carriers by use of a hedstrom file in case of retreatment. The groove is wider in the coronal part than in the middle part of the carrier, and the slices to be subjected to the push-out test were therefore prepared from the middle part of the obturator in order to reduce to a minimum the surface alteration caused by the presence of the groove. In addition, the taper of the carrier in the tested endodontic obturators was 0.04 mm; this taper would not have excessively influenced the results, considering that the tested slices of the carriers were only 1 mm thick. Moreover, the push-out bond strength values were calculated according to the equation estimating the apical taper radius and coronal taper radius to obtain accurate results. The bond between the gutta-percha and the carrier of the experimental obturator was significantly higher than the one obtained with Thermafil and GuttaCore. This could be due to the micromechanical adhesion of the GP to the HA/PE fibres as the fibre-based carriers have irregular surfaces. Similarly, the push-out bond strength of the GuttaCore carrier was found to be significantly higher than that of the Thermafil carrier. Again the GuttaCore carrier appeared to offer better micromechanical retention than the Thermafil carrier, since the material used to fabricate the GuttaCore carrier is cross-linked GP and so it is unlikely that any chemical interaction would develop between this and the alpha-phase GP of the coating. The clinical relevance of the information we gathered on the push-out bond strength of the gutta-percha to the carriers is, however, somehow limited, considering that in a clinical context the carriers are heated before insertion into the root canal and this might result in the increased bond strength of the gutta-percha to the carrier.

The present research used a model that allowed for the assessment of the thickness of gutta-percha around the carrier material outside the root canal system using *μ*CT technology. Thermafil and GuttaCore carriers are identical in shape and the gutta-percha used for the coating is also identical for the two systems; it was for this reason that only one of the two carrier types (GuttaCore) was used for the measurement of the thickness of the gutta-percha. *μ*CT technology has been used to assess root canal morphology [19, 20] and root canal obturation [15], quantitatively as well as qualitatively. Also, *μ*CT analysis has been used to evaluate root canal fillings and other dental materials in 3D reconstructions [21, 22]. In this study the *μ*CT scanner accurately quantified the distribution of gutta-percha around the carriers. In the GuttaCore carrier the thickness of GP was not uniformly distributed around the carrier and the carrier seemed to be positioned eccentrically. On the other hand, *μ*CT imaging showed that the thickness of GP on the HA/PE carrier was rather evenly distributed and the carrier was in the centre of GP. Further studies are required in order to investigate the push-out bond strength and the quality of the root canal fillings obtained using the most recently developed core-based systems particularly in curved canals. The present study concludes that the experimental HA/PE carrier exhibited the highest push-out bond strength and the new GuttaCore carriers showed a significant improvement in push-out strength compared with the Thermafil ones; thus the first hypothesis has been rejected. The second hypothesis has also been rejected because the thickness of gutta-percha coating of HA/PE was uniform in comparison to that of GuttaCore, which was not homogenous. It is expected that a good retention of gutta-percha around the carrier with a uniform thickness of gutta-percha coating would help prevent stripping of gutta-percha coating around the carrier material.

## Figures and Tables

**Table 1:** Endodontic obturators used in this study.

Product	Manufacturer	Material
GuttaCore#30	Dentsply/Tulsa	Cross-linked gutta-percha
Thermafil#30	Dentsply/Tulsa	Vectra
HA/PE	Experimental	Hydroxyapatite-polyethylene-strontium oxide

**Table 2:** Push-out bond strength of Thermafil, GuttaCore, and experimental carrier with gutta-percha coating. The data shows a statistically significant difference (*P* < 0.001) for the three different groups.

Product	Push-out bond strength in MPa
Thermafil	1.5 ± 0.63
GuttaCore	3.75 ± 0.75
Experimental HA/PE	6.84 ± 0.96
